# Investigating depression in multiple sclerosis: an Italian Delphi consensus on clinical manifestations, diagnosis and treatment

**DOI:** 10.3389/fpsyt.2025.1557335

**Published:** 2025-06-30

**Authors:** Antonio Bruno, Ettore Dolcetti, Pietro Annovazzi, Marinella Clerico, Eleonora Cocco, Antonella Conte, Girolama Alessandra Marfia, Marco Salvetti, Valentina Tomassini, Valentina Torri Clerici, Rocco Totaro, Ferdinando Nicoletti, Diego Centonze

**Affiliations:** ^1^ Neurology Unit - IRCCS Neuromed, Pozzilli (IS), Italy; ^2^ Neuroimmunology Unit - Multiple Sclerosis Centre ASST Valle Olona - Gallarate Hospital, Gallarate (VA), Italy; ^3^ Clinical and Biological Sciences Department, University of Torino, Torino, Italy; ^4^ University of Cagliari, Department of Medical Science and Public Health, Centro Sclerosi Multipla, Cagliari, Italy; ^5^ Department of Human Neurosciences, Sapienza, University of Rome, Rome, Italy; ^6^ Department of Systems Medicine, Tor Vergata University, Rome, Italy; ^7^ Multiple Sclerosis Clinical and Research Unit, Tor Vergata University Hospital, Rome, Italy; ^8^ Centre for Experimental Neurological Therapies (CENTERS), Department of Neurosciences, Mental Health and Sensory Organs, Sapienza University of Rome, Rome, Italy; ^9^ Institute for Advanced Biomedical Technologies (ITAB), Department of Neurosciences, Imaging and Clinical Sciences, University G. d’Annunzio of Chieti-Pescara, Chieti, Italy; ^10^ IRCCS Istituto Neurologico C. Besta, Neuroimmunology Unit, Milan, Italy; ^11^ Demyelinating Disease Center, Department of Neurology, San Salvatore Hospital, L’Aquila,, Italy; ^12^ Department of Physiology and Pharmacology, Sapienza University of Rome, Rome, Italy

**Keywords:** multiple sclerosis, depression, antidepressant therapy, disease-modifying therapy, DSM-5, disease modifying symptomatic treatment

## Abstract

**Background:**

In multiple sclerosis (MS), depression represents one of the most frequent psychiatric comorbidities, significantly impacting patients’ quality of life and disease progression. However, the diagnosis and management of depression in MS remain challenging due to overlapping symptoms and the lack of specific treatment guidelines. This Delphi study aims to achieve a shared consensus among Italian MS experts regarding the clinical manifestations, diagnosis, and treatment of depression in MS.

**Methods:**

An online Delphi survey with 35 questions covering the epidemiology, clinical features, diagnostic criteria, and treatment of depression in MS was anonymously administered to a panel of 51 expert neurologists across Italy. The consensus was based on a two-round Delphi process.

**Results:**

Consensus was reached on 100% of the statements. Positive consensus was achieved on 90.6% of the statements, while 9.4% reached negative consensus. Key findings include the strong link between depression and MS, with depressive symptoms often preceding MS onset. The panel agreed that the DSM-5 diagnostic criteria should be adapted to capture the specific mood disturbances seen in MS. Regarding treatment, antidepressants were widely prescribed, but concerns about their efficacy in the MS population remain. Non-pharmacological interventions, such as cognitive behavioral therapy (CBT), were considered essential components of comprehensive care.

**Conclusions:**

This Delphi study highlights the need for tailored diagnostic tools and integrated treatment approaches for managing depression in MS. Further studies are required to refine guidelines for the use of antidepressants and explore the role of disease-modifying therapies (DMTs) in treating depression in this population.

## 1 Introduction

Multiple sclerosis (MS) is a chronic neuroinflammatory disease affecting about 2.8 million people globally, known primarily for physical symptoms including motor impairment, fatigue, and sensory disturbances (1). Advances in disease-modifying therapies (DMTs) have improved physical symptom control, allowing clinicians to focus more on secondary symptoms, such as depression. Depressive symptoms have a lifetime prevalence of 50% in MS patients and are often underreported. Despite their high prevalence and the significant impact on quality of life (QoL), the diagnosis and management of depression in MS is still a matter of debate among clinicians (2). Depressive symptoms such as fatigue, pain, cognitive dysfunction, and sleep disturbances may be part of MS condition and this could complicate diagnosis. This also frequently leads to underdiagnosis and delays in treatment (3, 4). Untreated depression in MS is strongly associated with poor outcomes, including increased disability and reduced adherence to pharmacological treatments and rehabilitation programs (5–7). Despite the existence of multiple consensus guidelines in the literature, there is still no global agreement on managing depressive disorders in MS (8–10). Current treatment approaches rely primarily on antidepressants, though non-pharmacological options such as cognitive behavioral therapy (CBT) are increasingly recognized as essential components of comprehensive care (2, 11, 12). Interestingly, no consensus has yet been established on the optimal choice of antidepressant for MS, the recommended duration of treatment, or the primary symptoms that should be prioritized. This is especially important given that the pharmacological profile of antidepressants can either alleviate or exacerbate various symptoms commonly associated with MS. This Delphi study aims to establish a unified perspective among Italian MS specialists on the diagnosis and management of depression in MS, offering valuable insights to inform future clinical practice and research.

## 2 Methods

### 2.1 Consensus determination through Delphi methodology

This study employed a Delphi methodology to achieve expert consensus on topics related to depression and MS. The Delphi process is a structured, iterative method designed to converge expert opinions through successive rounds of anonymous feedback and voting. Participants were invited to express their agreement or disagreement on predefined statements using a 5-point Likert scale, where 1 indicated “strongly disagree,” 2 denoted “disagree,” 3 represented “neutral,” 4 signified “agree,” and 5 corresponded to “strongly agree.” These thresholds (66% for agreement and 75% for disagreement) were based on commonly adopted criteria in Delphi studies within clinical research, ensuring balance between inclusivity and robustness of expert consensus. Consensus was defined as an agreement level of 66%, calculated as the combined percentage of participants selecting scores of 4 or 5. Conversely, disagreement was defined when 75% of responses combined scores of 1 or 2. Statements that did not meet either threshold were considered as lacking consensus. Multiple rounds of voting were performed when necessary to refine responses and attempt to achieve consensus.

### 2.2 Statements preparation

An Italian Steering Committee, composed of experts in neurology, psychiatry, and related fields, developed the statements for this study. Selection of Steering Committee members was based on their clinical expertise and prior contributions to the management of depression in patients with MS. Specifically, the selection of Steering Committee members was based on publication record in MS and depression, and recognized roles in national clinical networks. Statements were derived from a systematic review of the literature and practical insights into unmet clinical needs. The Steering Committee finalized a total of 32 statements, distributed across four primary topics: Depression and MS (5 statements); Clinical manifestations of depression in MS patients (9 statements); Diagnostic criteria for depression in MS (3 statements); Treatment options for depression in MS (15 statements). Statements were based on a structured review of available literature and unmet clinical needs, as perceived by the Steering Committee. No formal pilot testing was conducted prior to dissemination to the expert panel. All statements were drafted in Italian and validated through a consensus-driven process involving external reviewers for clarity and relevance.

### 2.3 Expert panel

The Delphi panel included 51 healthcare professionals with expertise in neurology, psychiatry, and clinical psychology. The participants were selected from hospitals, university centers, and IRCCS (Istituti di Ricovero e Cura a Carattere Scientifico) to ensure a broad representation of clinical settings. The gender distribution was 39% male (n = 20) and 61% female (n = 31). Regarding years of clinical experience, the panel included experts ranging from early-career professionals to senior clinicians with over 30 years of experience. Geographically, participants were distributed across Italy, encompassing northern, central and southern regions, to capture potential regional variations in clinical practice. The types of institutions represented were: Territorial hospitals: 45% (n = 23); University hospitals: 47% (n = 24); IRCCS: 8% (n = 4). Voting sessions were conducted remotely, ensuring anonymity to reduce bias. Data were collected and analyzed independently by the study coordinators to maintain objectivity.

## 3 Results

The Delphi study on depression in MS yielded consensus on all examined statements with a consensus threshold set at 66; a positive agreement was reached in most cases. This study involved 51 Italian experts and focused on clinical manifestations, diagnosis, and treatment of depression in MS.

### 3.1 Depression and MS

**Figure 1:**
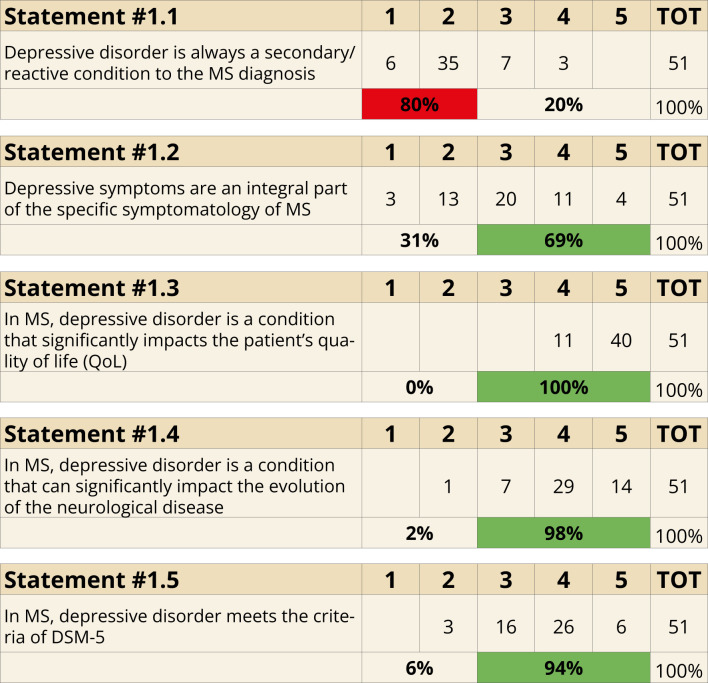
Depression and MS (Statements 1.1-1.2-1.3-1.4-1.5).

The panel established that depression in MS is not solely a reactive condition. While 80% (Statement 1.1) of participants disagreed with the idea that depressive symptoms are always secondary to MS diagnosis, 69% (Statement 1.2) agreed that depressive symptoms are intrinsic to the disease’s clinical presentation. Consensus was unanimous (100%) that depression significantly impacts patients’ QoL (Statement 1.3) and that it influences disease progression (98%, Statement 1.4). The panel also largely supported (94%, Statement 1.5) the application of the diagnostic and statistical manual of mental disorders (DSM)-5 criteria for diagnosing depressive disorders in MS (**Figure 1**).

### 3.2 Clinical manifestations

**Figure 2:**
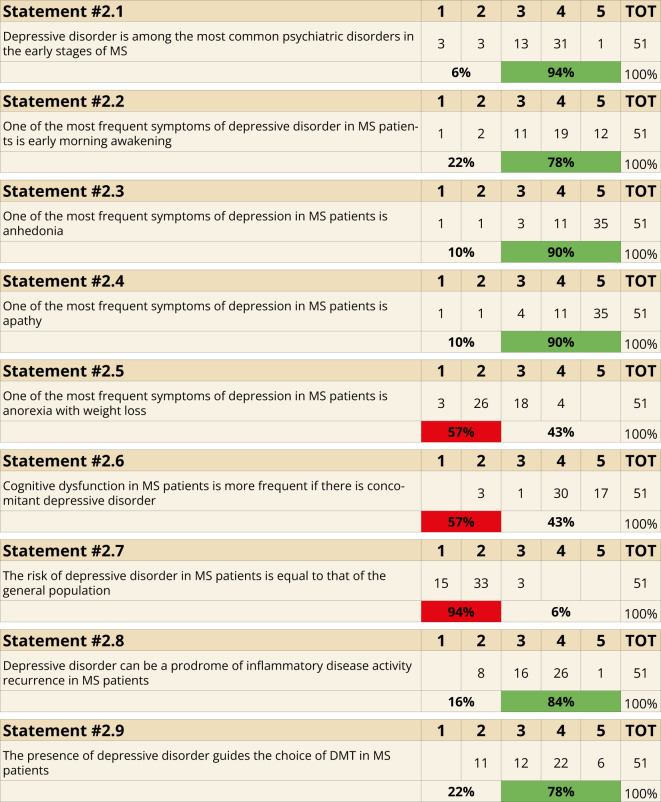
Clinical Manifestations (Statements 2.1-2.2-2.3-2.4-2.5-2.6-2.7-2.8-2.9).

Depression was identified as a common psychiatric comorbidity during the early stages of MS (94%, Statement 2.1). Specific symptoms frequently associated with depression in MS included early morning awakening (78%, Statement 2.2), anhedonia (90%, Statement 2.3), and apathy (90%, Statement 2.4). Conversely, anorexia and weight loss were not recognized as frequent symptoms, as only 43% (Statement 2.5) supported this association. Cognitive dysfunction was acknowledged as being more prevalent when depression is present in MS patients (94%, Statement 2.6). The risk of developing depression in MS patients was considered significantly higher than in the general population (94%, Statement 2.7). Furthermore, depressive symptoms were identified as potential prodromal indicators of disease activity (84%, Statement 2.8), and their presence was seen as a critical factor influencing the selection of disease-modifying treatments (78%, Statement 2.9) (**Figure 2**).

### 3.3 Diagnostic criteria

**Figure 3:**
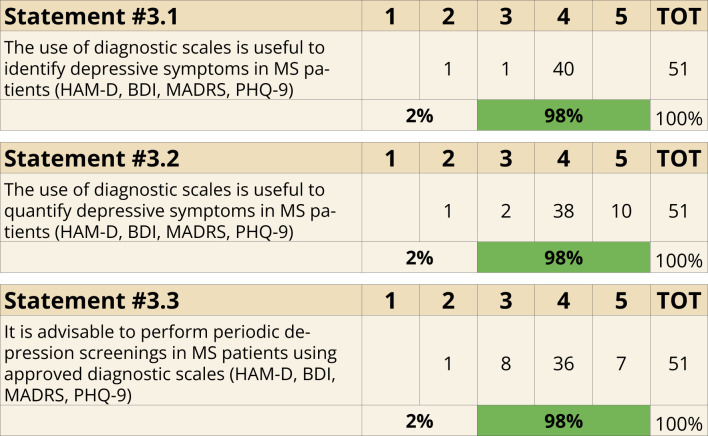
Diagnostic criteria (Statements 3.1-3.2-3.3).

The importance of diagnostic tools was strongly emphasized, with 98% of the panel agreeing on the utility of scales like HAM-D, BDI, MADRS, and PHQ-9 for identifying (Statement 3.1) and quantifying (Statement 3.2) depressive symptoms. Regular screenings for depressive symptoms in MS patients using validated scales were also strongly recommended (98%, Statement 3.3) (**Figure 3**).

### 3.4 Treatment

Regarding treatment approaches, the optimization of MS-specific therapies was recognized as a primary step in managing depressive symptoms (69%, Statement 4.1). Antidepressants that minimize weight gain (98%, Statement 4.2) and sexual dysfunction (100%, Statement 4.3) were considered ideal. Furthermore, the selection of antidepressants based on their impact on urinary function was supported by 86% (Statement 4.4). However, the selection of antidepressants with anti-inflammatory properties received only 59% support (Statement 4.5). Cognitive symptoms (96%, Statement 4.6), anhedonia (98%, Statement 4.7), fatigue (100%, Statement 4.8), and pain (98%, Statement 4.9) were identified as key factors guiding the choice of antidepressants.

**Figure 4:**
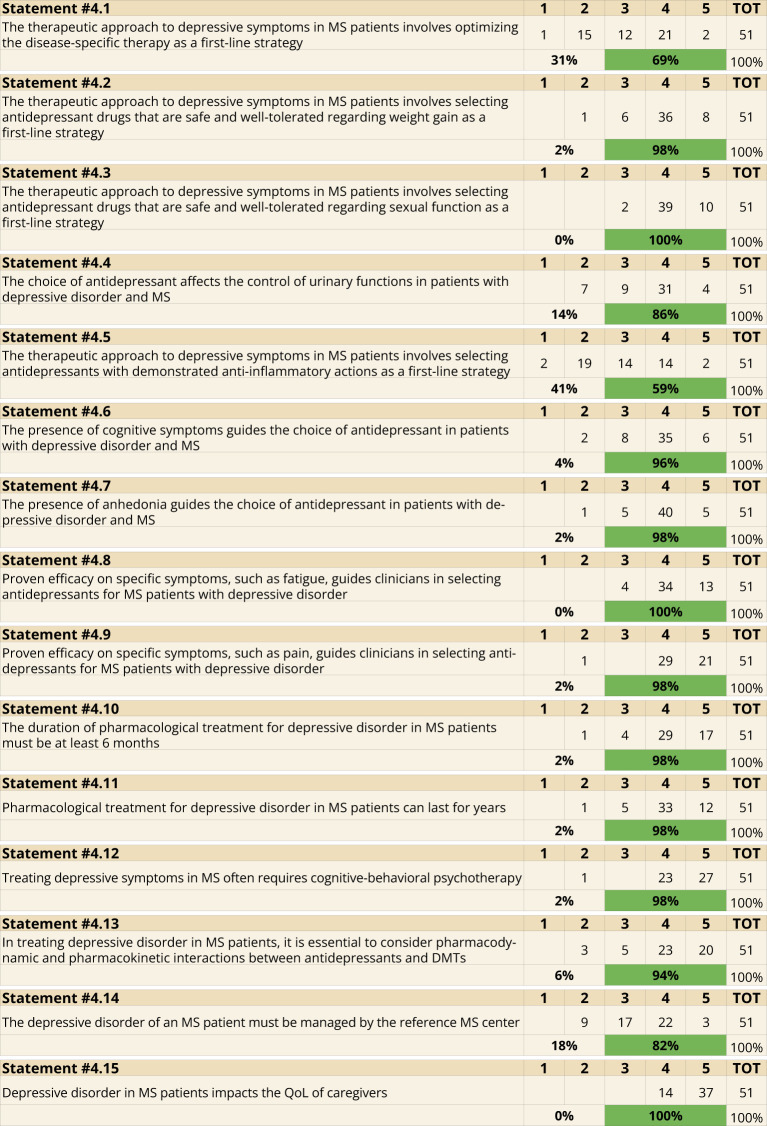
Treatment (Statements 4.1-4.2-4.3-4.4-4.5-4.6-4.7-4.8-4.9-4.10-4.11-4.12-4.13-4.14-4.15).

For pharmacological treatments, a minimum duration of six months was recommended by 98% (Statement 4.10), with potential extension for years if needed (Statement 4.11). Non-pharmacological interventions, particularly CBT, were highly endorsed (98%, Statement 4.12). Managing pharmacokinetic and pharmacodynamic interactions between antidepressants and disease-modifying therapies was also emphasized (94%, Statement 4.13). Centralizing the management of depression in MS reference centers was advocated by 82% (Statement 4.14). Lastly, all participants agreed that MS-related depression has a profound impact on caregivers’ QoL (Statement 4.15) (**Figure 4**).

## 4 Discussion

The objective of this Delphi consensus was to examine the challenges and strategies involved in managing depressive symptoms in MS, with the purpose of achieving a shared understanding among Italian expert neurologists. The following discussion delves into the main themes of the Delphi study, providing brief commentary on each item identified, alongside relevant insights from the latest literature.

### 4.1 Depression and MS

The nature of depressive syndrome in MS—whether it is ‘organic’ (an epiphenomenon resulting from increased inflammatory activity) or ‘reactive’ (a functional response to neurological and physical symptoms)—remains a topic of debate among MS experts (6, 13). In this Delphi consensus, 80% of participants agreed that depressive disorder in MS is not merely a reactive phenomenon occurring post-diagnosis (s 1.1). Supporting the organic theory, neuroinflammatory mediators such as tumor necrosis factor (TNF)-α and interleukin (IL)-6—integral to MS pathophysiology—have been associated with the severity of anxiety and depressive symptoms in MS patients (13) as well as in experimental autoimmune encephalomyelitis (EAE), an animal model of MS (14, 15). Depressive symptoms have also been linked to elevated levels of TNF-α and interferon (IFN)-γ during clinical relapses (13). Among the influence of central neuroinflammation, the evidence of morphological and functional alterations of gray and white matter in the brain of depressed MS patients is strongly supported by literature. Specific cerebral areas, such as frontal and temporal lobe, hippocampus, pre-frontal cortex, anterior cingulate and orbito-frontal cortex seems to be deeply involved in defining clinical phenotype of MS (16, 17). A reduced cortical thickness has been detected by high-filed 3T MRI sequences, and also a selective atrophy of specific subcortical areas, in particular thalamus and striatum, has been evidenced (16). Moreover, functional MRI (fMRI) showed alterations in functional connectivity between specific brain regions, including amygdala, frontal cortex, ACC and ventral striatum by BOLD-fMRI studies (18). In accordance with this theory, 69% of Delphi participants agree that depression could be considered an intrinsic symptom of MS (s 1.2). Nonetheless, nearly one-third of panelists remained cautious, observing that psychosocial stressors and the frequent convergence of fatigue, cognitive slowing and mood items can yield a chiefly “reactive” depression phenotype in routine practice, encouraging some clinicians to view depression as a comorbidity rather than a core disease feature (19). Psychiatric comorbidities, including depression, deeply impact QoL in MS patients, reducing resilience and adaptability to daily life and work environments (20). The totality of the panel (100%) agreed that depression in MS significantly impacts patients’ QoL (s 1.3). This is in line with current literature, as depression has been directly associated with lower QoL, independent of physical disability (19, 21–24). Interestingly, current literature suggests a correlation between the severity of depressive symptoms, disease course, and disability progression in MS, particularly in secondary progressive MS (SPMS) (25, 26). This aligns with the consensus of 98% of the panel, who affirmed that depressive disorder in MS could significantly impact disease progression (s 1.4). Furthermore, depressive symptoms have been linked to reduced adherence to both pharmacological treatments and rehabilitative programs, complicating disease management and potentially accelerating progression (5–7). Establishing criteria to accurately diagnose depression in MS is essential. While 94% of participants agreed that DSM-5 criteria align with depressive symptoms in MS (s 1.5), this is only partially consistent with existing literature. Other authors have suggested that DSM-5 criteria do not address the significant overlap between MS and depression symptoms, further complicating diagnosis (2). The Goldman Consensus recommends using scale-based assessments to identify clinically significant symptoms even if full depressive disorder criteria are not met (27). Other guidelines do not specify particular criteria or tools for diagnosing depression in MS (2, 28), suggesting that a global consensus is still lacking.

### 4.2 Clinical manifestations of depression in MS patients

In line with current literature, 94% of the panel affirmed that depressive disorder is the most frequent mood disorder at MS clinical onset (s 2.1). Defining specific symptoms of depression in MS remains an unmet goal for clinicians. The panel identified early morning awakening (78%, s 2.2), anhedonia (90%, s 2.3), and apathy (90%, s 2.4) as the most common symptoms — with the lower consensus on early-morning awakening probably reflecting the fact that, in MS, nocturia, pain and nocturnal spasms often disrupt sleep and can cause terminal insomnia even in the absence of depression (29). Literature also highlights a strong association between depression and anxiety in MS (6). Moreover, other depressive features in MS include feelings of helplessness, reduced social participation, and diminished enjoyment of activities (6, 25). Additionally, studies indicate that many MS patients present with a mixed atypical–melancholic major depressive disorder (MDD) subtype consistent with DSM-5 criteria, whereas a typical MDD phenotype does not clearly differentiate individuals with MS from those without the disease (4). In line with 57% of our panel, eating disorders such as anorexia, bulimia, and binge eating are not specifically associated with depressive disorder in MS (s 2.5), suggesting a more cautious approach in this context (30). Conversely, consistent with the views of 43% of our panel (s 2.5), some authors have linked depressive symptoms in MS with altered appetite and eating disorders. A likely explanation for this split is the scarcity and heterogeneity of data: a recent cohort study found that body-image dissatisfaction—and, by extension, clinically defined eating-disorder prevalence—does not exceed that of the general population after adjusting for depression (31), whereas smaller cross-sectional work has reported binge-eating and appetite dysregulation that correlate with depressive scores in MS patients (32). The majority (94%) of our panel considered cognitive dysfunction in MS more frequent if associated with concomitant depressive disorder (s 2.6). This is supported by MS literature that strongly associate depressive symptoms in MS with cognitive impairment (33) with particular involvement of attentive domain, executive function, processing speed, visual perception/organization (34) and verbal memory (35). A potential bidirectional link between depression and cognitive impairment in MS has been further substantiated (36). Structural and functional MRI studies reveal similar patterns of default mode network imbalance and functional disconnection in hippocampal regions across both conditions, further suggesting a potential bidirectional influence between them (36, 37). In our Delphi study, 94% of participants (s 2.7) disagreed with the notion that the risk of depressive disorder in MS is comparable to that of the general population. This consensus aligns with epidemiological data showing a high prevalence of depressive symptoms among MS patients, estimated to range from 15% to 45% depending on country and clinical setting (25, 38, 39). Further studies consistently report that the prevalence of major depression in MS is 2–4 times higher than in the general population (40), with a lifetime prevalence reaching up to 50% and notably lacking gender differences, a rate significantly higher than typically observed in MDD (17). Most participants agreed that depressive disorder in MS could serve as a prodromic indicator of disease activity (84%, s 2.8) and that its presence may guide therapeutic decisions regarding DMTs in MS patients (78%, s 2.9). Experimental and clinical data suggest that mood disorders in MS can precede relapse symptoms by several days to weeks (13, 41). Some DMTs, however, may adversely affect mood. Although emerging data suggest that highly effective DMTs can influence the burden of mood comorbidities in MS (42), the 78% agreement likely reflects the still-limited and sometimes conflicting evidence highlighting the need for prospective trials to define the true mood-modulating effects of individual therapies (43).

### 4.3 Diagnostic criteria of depressive disorder in MS patients

It is well-established that fewer than 30% of MS patients report depressive symptoms as they occur, potentially due to beliefs that depression is inevitable or due to practical barriers to seeking treatment (44, 45). Additionally, a recent Australian study noted reluctance among some MS patients to receive an additional diagnosis beyond MS (2). In view of this, the use of screening tools becomes essential for identifying depressive disorders that patients may not openly report. This aligns with the 98% consensus of our Delphi panel, which underscored the importance of clinical scales—such as HAM-D, BDI and MADRS—for identifying (s 3.1) and quantifying (s 3.2) depressive symptoms in MS. Consistent with this finding, both the Goldman Consensus (27) and the 2014 American Academy of Neurology guidelines (46) recommend routine screening with instruments like the PHQ-9 or BDI, although they differ on optimal intervals and cut-off values. Tool selection remains debated: three guidelines (25, 27, 46), favor the Beck Depression Inventory, and the Goldman panel proposes a BDI threshold of 13, whereas the AAN warns that this cut-off may miss up to 30% of cases and offers no alternative (46). Additionally, the German MS Therapy Consensus Group (MSTCG) generally supports screening but does not specify tools or methods (47). In recent years, the development of specific clinical scales to detect depressive symptoms in MS patients has been assessed. The Multiple Sclerosis Depression Rating Scale (MSDRS) represents one of the most effective in defining and monitoring clinical symptoms of depression in MS patients, based on a semi-structured interview to patients divided into 9 section, investigating respectively depressed mood, feelings of guilt, thoughts of death, vegetative disorders, apathy and loss of interest, anxiety, hyperemotionalism, emotional reactivity, and diurnal mood variations, assigning them a score variable from positive to negative pole and correcting data in relation to the presence of fatigue (48). Rather than other scales, MSRDS provides a clearer evaluation of psychiatric comorbidities taking into account physical symptoms experimented by MS patients. According to AAN guidelines (46) and the Canadian Network for Mood and Anxiety Treatments (CANMAT) task force (28), 98% of participants also recommended periodic screening for depressive symptoms in MS patients using validated clinical scales (s 3.3). Existing guidelines, advocate for active re-evaluation in MS patients with a history of mild or transient depressive episodes. According to AAN guidelines (46) and recently published clinical practice guidelines (2), annual or each visit screening is particularly recommended for those on antidepressant therapy or with a history of hypomanic or manic symptoms, given the higher prevalence of bipolar disorder in MS (2, 28). Considering these aspects, the need of implementing the use of clinical scales in diagnostic depression in MS patients should be addressed. The presence of dedicated figures, such as neuropsychologists, in second-level healthcare settings for the treatment of MS patients could help to standardize the use of quantitative scales, supporting diagnosis of depression in MS, after a first step evaluation. In this regard, it is well known that interdisciplinary contexts ameliorate outcomes in the treatment of MS patients (49).

### 4.4 Treatment of depression in MS patients

The relationship between MS DMTs and depression introduces additional complexity to treatment decisions. For example, some DMTs may exacerbate depressive symptoms, while others appear neutral or even beneficial for mood (43). In this Delphi study, 69% of participants agreed that the first-line approach to managing depressive symptoms in MS patients should emphasize optimizing MS-specific treatments (s 4.1). This partial consensus reflects the Goldman Consensus Statement (27) and the 2014 AAN guideline (46), which recognize depression as a frequent comorbidity in MS but do not classify it as an “intrinsic” manifestation of the disease. The limited agreement also mirrors the uneven evidence on mood effects of individual DMTs: studies on interferon-β, for example, report neutral, beneficial, or even adverse impacts on depressive symptoms (50, 51). By contrast, other DMTs—such as dimethyl fumarate, teriflunomide, fingolimod, natalizumab and alemtuzumab—have shown potential in alleviating depressive symptoms in MS likely by modulating inflammatory pathways (50, 52–54) and influencing the neurobiological mechanisms underlying mood disorders (55). The specific treatment of depression in MS remains a topic of debate, with both pharmacological and non-pharmacological options being recommended. Regarding pharmacological treatment, the majority of the panel (98%) agreed that the selection of antidepressants should prioritize agents that are safe and well-tolerated, with minimal impact on weight gain (s 4.2). In this regard, notable options are vortioxetine, fluoxetine, agomelatine (56) and venlafaxine (57). The entire panel of Delphi participants agrees that the choice of antidepressants should prioritize drugs that are well-tolerated in terms of sexual functioning (s 4.3). Sexual dysfunction is a frequent cause of non-adherence to antidepressant treatment, especially among young men (58). Notably, several selective serotonin reuptake inhibitors (SSRIs), serotonin-norepinephrine reuptake inhibitors (SNRIs), and tricyclic antidepressants (TCAs) commonly used in the clinical practice, such as duloxetine, venlafaxine, clomipramine, and paroxetine, are associated with sexual dysfunction (59). Conversely, newer SSRIs antidepressants, like vortioxetine (60), and other antidepressants, such as mirtazapine and bupropion, are associated with a lower risk of sexual side effects (58). Another priority shared by the 86% of participants was on the impact of antidepressants on urinary function (s 4.4). Urinary dysfunction is often linked to the anticholinergic or adrenergic side effects of antidepressants. A recent systematic review showed that TCAs and SNRIs carry a higher risk of voiding issues, such as hesitancy or chronic urinary retention, compared to SSRIs. Specifically, duloxetine has been associated with an increased risk of urinary retention. Alternatively, duloxetine has demonstrated effectiveness in MS patients with overactive bladder syndrome, which may include urinary incontinence (61). Additionally, it may indirectly benefit female sexual function by alleviating stress incontinence (62). Current literature highlights an anti-inflammatory role for several antidepressants—SSRIs reduce TNF-α and IL-6 and dampen microglial activation, whereas TCAs lower other pro-inflammatory cytokines and raise IL-10 (17). Yet only 59% of Delphi panelists recommended giving priority to antidepressants with these putative immunomodulatory effects (s 4.5). The limited consensus likely stems from clinical uncertainty also derived from current guidelines. AAN (46) and CANMAT (28) state that evidence for disease-modifying effects of antidepressants is still insufficient. Moreover, the Goldman statement (27) does not mention anti-inflammatory selection. Human evidence is still confined to small, often conflicting observational or exploratory studies that only hint at a disease-modifying potential of SSRIs—both in preventing relapses (63, 64) and in slowing disability progression in MS (17, 65). Vortioxetine has likewise been proposed as a disease-modifying symptomatic treatment (DMST), having reduced microglial toxicity and preserved neuronal integrity in EAE models (60). The majority (96%) of participants indicated that cognitive symptoms influence the choice of antidepressant in MS patients with depression (s 4.6). Although no specific antidepressant is currently endorsed for treating cognitive dysfunction in MS, vortioxetine has shown promise in enhancing processing speed and overall cognition, likely due to its unique pharmacodynamic profile (60, 66). In addition to cognitive symptoms, 98% of the panel also prioritized the role of anhedonia in guiding antidepressant selection (s 4.7). Traditional SSRIs and SNRIs show limited efficacy in treating anhedonia, whereas newer options like agomelatine (67) and ketamine (68) have shown some benefit. Vortioxetine is similarly proposed as a promising treatment for emotional blunting (69, 70), potentially enhancing overall functioning, motivation, and energy (60). Fatigue also emerged as a key factor, with unanimous agreement that its presence should guide antidepressant choice (s 4.8). Though evidence is limited, bupropion has shown potential benefits in managing fatigue in depressed patients (71) and appears to be more effective than SSRIs in this regard (72). Pain was also highlighted as a symptom that guides antidepressant choice, with 98% of participants emphasizing its importance (s 4.9). Neuropathic pain, affecting an estimated 4% to 26% of MS patients (65, 73), is commonly managed with duloxetine, which is FDA-approved for diabetic neuropathy and has shown efficacy in MS-related neuropathic pain at doses of 30–120 mg daily (74). Venlafaxine may also be an option for pain relief in MS, though evidence is mixed, with some studies supporting its use in neuropathic pain and migraine, a common MS comorbidity tied to worsened symptoms, including depression (75). Emerging research suggests that vortioxetine in depressed patients might benefit also chronic neuropathic pain by selectively modulating 5-HT3 and 5-HT7 receptors, presenting potential advantages over traditional SNRIs (60, 76). In our Delphi, 98% of participants agreed on the importance of incorporating non-pharmacological interventions, particularly CBT, in managing depressive symptoms in MS (s 4.12). CBT is supported by evidence for improving mood, lessening fatigue, and strengthening coping skills (11). It is well known that CBT as structured intervention is not available in all clinical contexts. Even if nationwide studies for mapping regional distribution of neuropsychological assessment have not been conducted yet, in Italian MS centers availability of CBT appears not homogeneous (77). Although it could represent a limitation referring to the high percentage of consensus reached in our Delphi, the implementation of dedicate personnel in MS teams should be considered an unmet need to achieve in order to revise therapeutic approach in this direction. Consistently, the AAN guideline awards Level C evidence to a telephone-delivered CBT program for MS patients, underscoring its usefulness when in-person therapy is not feasible (46). Beyond the pharmacological and psychological treatment of depression in MS, non-invasive brain stimulation (NIBS) could contribute to ameliorate depressive symptoms (78, 79). We know from literature that stimulation by intermittent-theta burst stimulation (iTBS) of orbito-frontal cortex in patients with depressive disorder shows efficacy in recovering from acute phases (80). Moreover, anodal tDCS of the left dorsolateral prefrontal cortex (DLPFC) (with right orbitofrontal cathode) seems to have a positive outcome in the treatment of major depressive episode without drug resistance (81). Although specific guidelines for the employment of NIBS in treating MS related depression have not been evaluated yet, an extension of protocols currently in use in depressive disorder could be considered. Regarding pharmacological treatment, 98% of participants recommended a minimum six-month duration for antidepressant use, with extension as needed (s 4.10, s 4.11). To optimize depression management, 94% of participants also emphasized the importance of monitoring pharmacodynamic and pharmacokinetic interactions between antidepressants and DMTs (s 4.13) as suggested by previous Guidelines (2, 28). Additionally, 82% of the panel agreed that depressive symptoms should be managed by an MS reference center, ensuring a cohesive treatment plan that addresses the complex pathophysiology of depression in MS, facilitating more tailored care (s 4.14). Finally, all participants (100%) acknowledged the significant impact of MS-related depression on the QoL of caregivers (s 4.15). Caring for an MS patient with depression can be demanding, emphasizing the importance of support systems and resources for both patients and caregivers. This need is similarly highlighted in other chronic conditions, such as Alzheimer’s disease (82), non-malignant chronic illnesses (including neurological conditions, coronary heart disease, and kidney disease) (83), and psychiatric conditions (59). This aspect underscores the necessity of integrated care strategies that address the well-being of both patients and their caregivers.

## 5 Study limitations

This Delphi study, while robust in its methodology, has several limitations that warrant consideration. First, the expert panel was composed exclusively of Italian clinicians, which may limit the generalizability of findings to other healthcare systems or cultural contexts. Second, the different clinical regional settings in which neurologists usually operate could represents a limitation of applicability of standardized Delphi consensus. The application of shared clinical guidelines could prove to be a demanding, but at the same time exciting challenge in relation to the variability of Italian healthcare system. Third, while the Delphi methodology facilitates iterative refinement, the absence of patient perspectives precludes a holistic understanding of depression impact on those living with multiple sclerosis. Additionally, the reliance on predefined statements may have constrained the scope of discussion, potentially overlooking nuanced aspects of clinical practice. Finally, the study outcomes remain theoretical and lack direct empirical validation, underscoring the necessity for future clinical studies to confirm the practical applicability of the recommendations. Moreover, this manuscript reports the descriptive analysis of response frequencies per statement. No inferential statistical analyses (e.g., subgroup comparisons) were planned or performed, as they were beyond the scope of the consensus approach adopted. This limits the interpretation of potential associations between respondent characteristics and voting behavior. Qualitative feedback was not systematically collected as part of the Delphi process. This limited the opportunity to explore the motivations behind specific voting patterns or divergent opinions.

## 6 Conclusions

This Delphi consensus highlights key insights into the diagnosis and management of depression in MS for an Italian cohort of neurologists. Participants affirmed the high prevalence and early onset of depressive symptoms in MS, significantly impacting both patients’ and caregivers’ QoL. Moreover, they emphasized depression as a core symptom of MS, highlighting its shared pathophysiology with the disease and the potential to precede MS onset by several years. Regarding the diagnostic tools, while DSM-5 criteria were generally endorsed, participants recommended additional and repetitive scale-based assessments to more accurately capture MS-specific depressive symptoms. In terms of pharmacological treatment, most participants emphasized prioritizing antidepressants with minimal side effects on weight, sexual function, and urinary health, though consensus on the optimal choice remains to be established. Newer antidepressants, such as vortioxetine, show promise in addressing cognitive symptoms and reducing central neuroinflammation. Non-pharmacological treatments, especially CBT, received strong support, along with a recommendation for integrating depression management within specialized MS centers for cohesive care. In conclusion, this Delphi study of Italian clinicians highlights the importance of a global consensus based on the evidence and further suggests interesting areas for future research.

## Data Availability

The raw data supporting the conclusions of this article will be made available by the authors, without undue reservation.

## References

[1] Jakimovski D Bittner S Zivadinov R Morrow SA Benedict RH Zipp F. Multiple sclerosis. Lancet. (2024) 403:183–202. doi: 10.1016/S0140-6736(23)01473-3 37949093

[2] McIntosh GE Liu ES Allan M Grech LB. Clinical practice guidelines for the detection and treatment of depression in multiple sclerosis. Neurol Clin Pract. (2023) 13. doi: 10.1212/CPJ.0000000000200154 PMC10132261 37124459

[3] Patten SB Marrie RA Carta MG. Depression in multiple sclerosis. Int Rev Psychiatry. (2017) 29:463–72. doi: 10.1080/09540261.2017.1322555 28681616

[4] Rodgers S Manjaly ZM Calabrese P Steinemann N Kaufmann M Salmen A. The effect of depression on health-related quality of life is mediated by fatigue in persons with multiple sclerosis. Brain Sci. (2021) 11:751. doi: 10.3390/brainsci11060751 34198920 PMC8227168

[5] Marrie R Horwitz R Cutter G Tyry T Campagnolo D Vollmer T. The burden of mental comorbidity in multiple sclerosis: frequent, underdiagnosed, and undertreated. Multiple Sclerosis J. (2009) 15:385–92. doi: 10.1177/1352458508099477 19153176

[6] Feinstein A Magalhaes S Richard JF Audet B Moore C. The link between multiple sclerosis and depression. Nat Rev Neurol. (2014) 10:507–17. doi: 10.1038/nrneurol.2014.139 25112509

[7] Binzer S McKay KA Brenner P Hillert J Manouchehrinia A. Disability worsening among persons with multiple sclerosis and depression. Neurology. (2019) 93. doi: 10.1212/WNL.0000000000008617 PMC6937491 31704791

[8] Fiest KM Walker JR Bernstein CN Graff LA Zarychanski R Abou-Setta AM. Systematic review and meta-analysis of interventions for depression and anxiety in persons with multiple sclerosis. Mult Scler Relat Disord. (2016) 5:12–26. doi: 10.1016/j.msard.2015.10.004 26856938

[9] Stepleman LM Penwell-Waines LM Rollock M Casillas RS Brands T Campbell J. Routine depression screening in an MS clinic and its association with provider treatment recommendations and related treatment outcome. J Clin Psychol Med Settings. (2014) 21:347–55. doi: 10.1007/s10880-014-9409-0 25194308

[10] Solaro C Bergamaschi R Rezzani C Mueller M Trabucco E Bargiggia V. Duloxetine is effective in treating depression in multiple sclerosis patients. Clin Neuropharmacol. (2013) 36:114–6. doi: 10.1097/WNF.0b013e3182996400 23783007

[11] Hind D Cotter J Thake A Bradburn M Cooper C Isaac C. Cognitive behavioral therapy for the treatment of depression in people with multiple sclerosis: a systematic review and meta-analysis. BMC Psychiatry. (2014) 14:5. doi: 10.1186/1471-244X-14-5 24406031 PMC3890565

[12] Grossman P Kappos L Gensicke H D’Souza M Mohr DC Penner IK. MS quality of life, depression, and fatigue improve after mindfulness training. Neurology. (2010) 75:1141–9. doi: 10.1212/WNL.0b013e3181f4d80d PMC3463050 20876468

[13] Rossi S Studer V Motta C Polidoro S Perugini J Macchiarulo G. Neuroinflammation drives anxiety and depression in relapsing-remitting multiple sclerosis. Neurology. (2017) 89:1338–47. doi: 10.1212/WNL.0000000000004411 28842450

[14] Gentile A Fresegna D Federici M Musella A Rizzo FR Sepman H. Dopaminergic dysfunction is associated with IL-1β-dependent mood alterations in experimental autoimmune encephalomyelitis. Neurobiol Dis. (2015) 74:347–58. doi: 10.1016/j.nbd.2014.11.022 25511803

[15] Haji N Mandolesi G Gentile A Sacchetti L Fresegna D Rossi S. TNF-α-mediated anxiety in a mouse model of multiple sclerosis. Exp Neurol. (2012) 237:296–303. doi: 10.1016/j.expneurol.2012.07.010 22836148

[16] Suh JS Schneider MA Minuzzi L MacQueen GM Strother SC Kennedy SH. Cortical thickness in major depressive disorder: A systematic review and meta-analysis. Prog Neuropsychopharmacol Biol Psychiatry. (2019) 88:287–302. doi: 10.1016/j.pnpbp.2018.08.008 30118825

[17] Bruno A Dolcetti E Rizzo FR Fresegna D Musella A Gentile A. Inflammation-associated synaptic alterations as shared threads in depression and multiple sclerosis. Front Cell Neurosci. (2020) 14. doi: 10.3389/fncel.2020.00169 PMC7324636 32655374

[18] Iwabuchi SJ Krishnadas R Li C Auer DP Radua J Palaniyappan L. Localized connectivity in depression: A meta-analysis of resting state functional imaging studies. Neurosci Biobehav Rev. (2015) 51:77–86. doi: 10.1016/j.neubiorev.2015.01.006 25597656

[19] Plow M Gunzler DD. Disentangling self-reported fatigue, depression, and cognitive impairment in people with multiple sclerosis. Mult Scler Relat Disord. (2022) 61:103736. doi: 10.1016/j.msard.2022.103736 35405560

[20] Mallucci G Monti MC Ponzio M Borrelli P Montomoli C Bergamaschi R. Impact of multiple sclerosis comorbidities on quality of life and job activity. Multiple Sclerosis J. (2024) 30:1047–55. doi: 10.1177/13524585241260550 38912795

[21] Ochoa-Morales A Hernández-Mojica T Paz-Rodríguez F Jara-Prado A Trujillo-De Los Santos Z Sánchez-Guzmán MA. Quality of life in patients with multiple sclerosis and its association with depressive symptoms and physical disability. Mult Scler Relat Disord. (2019) 36:101386. doi: 10.1016/j.msard.2019.101386 31520986

[22] Hayter AL Salkovskis PM Silber E Morris RG. The impact of health anxiety in patients with relapsing remitting multiple sclerosis: Misperception, misattribution and quality of life. Br J Clin Psychol. (2016) 55:371–86. doi: 10.1111/bjc.2016.55.issue-4 26806805

[23] Ribbons K Lea R Schofield PW Lechner-Scott J. Anxiety levels are independently associated with cognitive performance in an Australian multiple sclerosis patient cohort. J Neuropsychiatry Clin Neurosci. (2017) 29:128–34. doi: 10.1176/appi.neuropsych.16050085 27899051

[24] Yadav V Hegde S Netravathi M Philip M Cranberg L. Impact of cognitive and psychological functions in relapsing–remitting multiple sclerosis: A cross-sectional study. Ann Indian Acad Neurol. (2024). doi: 10.4103/aian.aian_434_24 PMC11575873 39388406

[25] Solaro C Trabucco E Signori A Martinelli V Radaelli M Centonze D. Depressive symptoms correlate with disability and disease course in multiple sclerosis patients: an Italian multi-center study using the beck depression inventory. PloS One. (2016) 11:e0160261. doi: 10.1371/journal.pone.0160261 27632167 PMC5025048

[26] Ajdacic-Gross V Steinemann N Horváth G Rodgers S Kaufmann M Xu Y. Onset symptom clusters in multiple sclerosis: characteristics, comorbidities, and risk factors. Front Neurol. (2021) 12. doi: 10.3389/fneur.2021.693440 PMC8290323 34295301

[27] Schiffer RB Arnett P Ben-Zacharia A Benedict R Bobholz J Caruso L. The Goldman Consensus statement on depression in multiple sclerosis. Multiple Sclerosis J. (2005) 11:328–37. doi: 10.1191/1352458505ms1162oa 15957516

[28] Ramasubbu R Taylor VH Samaan Z Sockalingham S Li M Patten S. The Canadian Network for Mood and Anxiety Treatments (CANMAT) task force recommendations for the management of patients with mood disorders and select comorbid medical conditions. Ann Clin Psychiatry. (2012) 24:91–109. 22303525

[29] Zeng X Dorstyn DS Edwards G Kneebone I. The prevalence of insomnia in multiple sclerosis: A meta-analysis. Sleep Med Rev. (2023) 72:101842. doi: 10.1016/j.smrv.2023.101842 37660580

[30] Honarmand K Feinstein A. Validation of the Hospital Anxiety and Depression Scale for use with multiple sclerosis patients. Multiple Sclerosis J. (2009) 15:1518–24. doi: 10.1177/1352458509347150 19965520

[31] Stevens SD Thompson NR Sullivan AB. Prevalence and correlates of body image dissatisfaction in patients with multiple sclerosis. Int J MS Care. (2019) 21:207–13. doi: 10.7224/1537-2073.2018-066 PMC6819016 31680782

[32] Saul A Taylor B Blizzard L Simpson-Yap S Oddy W Probst Y. Associations between diet quality and depression, anxiety, and fatigue in multiple sclerosis. Mult Scler Relat Disord. (2022) 63:103910. doi: 10.1016/j.msard.2022.103910 35636273

[33] Margoni M Preziosa P Rocca MA Filippi M. Depressive symptoms, anxiety and cognitive impairment: emerging evidence in multiple sclerosis. Transl Psychiatry. (2023) 13:264. doi: 10.1038/s41398-023-02555-7 37468462 PMC10356956

[34] Sundgren M Maurex L Wahlin A Piehl F Brismar T. Cognitive impairment has a strong relation to nonsomatic symptoms of depression in relapsing-remitting multiple sclerosis. Arch Clin Neuropsychol. (2013) 28:144–55. doi: 10.1093/arclin/acs113 23291310

[35] Helminen J Jehkonen M. Relationship between neuropsychiatric symptoms and cognition in multiple sclerosis: A systematic review. Appl Neuropsychol Adult. (2024), 1–16. doi: 10.1080/23279095.2024.2403764 39325074

[36] Bonavita S Sacco R Esposito S d’Ambrosio A Della Corte M Corbo D. Default mode network changes in multiple sclerosis: a link between depression and cognitive impairment? Eur J Neurol. (2017) 24:27–36. doi: 10.1111/ene.2017.24.issue-1 27633185

[37] Rocca MA Pravatà E Valsasina P Radaelli M Colombo B Vacchi L. Hippocampal- DMN disconnectivity in MS is related to WM lesions and depression. Hum Brain Mapp. (2015) 36:5051–63. doi: 10.1002/hbm.v36.12 PMC6869286 26366641

[38] Amtmann D Kim J Chung H Bamer AM Askew RL Wu S. Comparing CESD-10, PHQ-9, and PROMIS depression instruments in individuals with multiple sclerosis. Rehabil Psychol. (2014) 59:220–9. doi: 10.1037/a0035919 PMC4059037 24661030

[39] Sacco R Santangelo G Stamenova S Bisecco A Bonavita S Lavorgna L. Psychometric properties and validity of Beck Depression Inventory II in multiple sclerosis. Eur J Neurol. (2016) 23:744–50. doi: 10.1111/ene.2016.23.issue-4 26782789

[40] Vilagut G Forero CG Barbaglia G Alonso J. Screening for depression in the general population with the center for epidemiologic studies depression (CES-D): A systematic review with meta-analysis. PloS One. (2016) 11:e0155431. doi: 10.1371/journal.pone.0155431 27182821 PMC4868329

[41] Mandolesi G Bullitta S Fresegna D Gentile A De Vito F Dolcetti E. Interferon-γ causes mood abnormalities by altering cannabinoid CB1 receptor function in the mouse striatum. Neurobiol Dis. (2017) 108:45–53. doi: 10.1016/j.nbd.2017.07.019 28757328

[42] Salter A Lancia S Kowalec K Fitzgerald KC Marrie RA. Investigating the prevalence of comorbidity in multiple sclerosis clinical trial populations. Neurology. (2024) 102. doi: 10.1212/WNL.0000000000209135 PMC11067694 38350062

[43] Longinetti E Frisell T Englund S Reutfors J Fang F Piehl F. Risk of depression in multiple sclerosis across disease-modifying therapies. Multiple Sclerosis J. (2022) 28:632–41. doi: 10.1177/13524585211031128 PMC8961249 34264143

[44] Bristow K Patten S. Treatment-seeking rates and associated mediating factors among individuals with depression. Can J Psychiatry. (2002) 47:660–5. doi: 10.1177/070674370204700708 12355678

[45] Sollom AC Kneebone II. Treatment of depression in people who have multiple sclerosis. Multiple Sclerosis J. (2007) 13:632–5. doi: 10.1177/1352458507072384 17548443

[46] Minden SL Feinstein A Kalb RC Miller D Mohr DC Patten SB. Evidence-based guideline: Assessment and management of psychiatric disorders in individuals with MS. Neurology. (2014) 82:174–81. doi: 10.1212/WNL.0000000000000013 PMC3897434 24376275

[47] Henze T Rieckmann P Toyka KV. Symptomatic treatment of multiple sclerosis. Eur Neurol. (2006) 56:78–105. doi: 10.1159/000095699 16966832

[48] Quaranta D Marra C Zinno M Patanella AK Messina MJ Piccininni C. Presentation and validation of the multiple sclerosis depression rating scale: A test specifically devised to investigate affective disorders in multiple sclerosis patients. Clin Neuropsychol. (2012) 26:571–87. doi: 10.1080/13854046.2012.668220 22428778

[49] Papeix C Gambotti L Assouad R Ewenczyck C Tanguy ML Pineau F. Evaluation of an integrated multidisciplinary approach in multiple sclerosis care: A prospective, randomized, controlled study. Mult Scler J Exp Transl Clin. (2015) 1. doi: 10.1177/2055217315608864 PMC5433398 28607706

[50] Kirzinger SS Jones J Siegwald A Crush AB. Relationship between disease-modifying therapy and depression in multiple sclerosis. Int J MS Care. (2013) 15:107–12. doi: 10.7224/1537-2073.2012-036 PMC3883027 24453772

[51] Comi G Patti F Rocca MA Mattioli FC Amato MP Gallo P. Efficacy of fingolimod and interferon beta-1b on cognitive, MRI, and clinical outcomes in relapsing–remitting multiple sclerosis: an 18-month, open-label, rater-blinded, randomized, multicenter study (the GOLDEN study). J Neurol. (2017) 264:2436–49. doi: 10.1007/s00415-017-8642-5 PMC5688215 29063244

[52] Montanari E Rottoli M Maimone D Confalonieri P Plewnia K Frigo M. A 12-month prospective, observational study evaluating the impact of disease-modifying treatment on emotional burden in recently-diagnosed multiple sclerosis patients: The POSIDONIA study. J Neurol Sci. (2016) 364:105–9. doi: 10.1016/j.jns.2016.02.047 27084226

[53] Gasim M Bernstein CN Graff LA Patten SB El-Gabalawy R Sareen J. Adverse psychiatric effects of disease-modifying therapies in multiple Sclerosis: A systematic review. Mult Scler Relat Disord. (2018) 26:124–56. doi: 10.1016/j.msard.2018.09.008 30248593

[54] Hunter SF Agius M Miller DM Cutter G Barbato L McCague K. Impact of a switch to fingolimod on depressive symptoms in patients with relapsing multiple sclerosis: An analysis from the EPOC (Evaluate Patient OutComes) trial. J Neurol Sci. (2016) 365:190–8. doi: 10.1016/j.jns.2016.03.024 27206905

[55] Gentile A Fresegna D Musella A Sepman H Bullitta S De Vito F. Interaction between interleukin-1β and type-1 cannabinoid receptor is involved in anxiety-like behavior in experimental autoimmune encephalomyelitis. J Neuroinflammation. (2016) 13:231. doi: 10.1186/s12974-016-0682-8 27589957 PMC5009553

[56] Solmi M Miola A Capone F Pallottino S Højlund M Firth J. Risk factors, prevention and treatment of weight gain associated with the use of antidepressants and antipsychotics: a state-of-the-art clinical review. Expert Opin Drug Saf. (2024) 23:1249–69. doi: 10.1080/14740338.2024.2396396 39225182

[57] Kamp CB Petersen JJ Faltermeier P Juul S Siddiqui F Moncrieff J. The risks of adverse events with venlafaxine for adults with major depressive disorder: a systematic review of randomized clinical trials with meta-analysis and Trial Sequential Analysis. Epidemiol Psychiatr Sci. (2024) 33:e51. doi: 10.1017/S2045796024000520 39440379 PMC11561525

[58] Rabinowitz MJ Li O Pil EH Eaton CK Kohn TP Haney NM. Antidepressant nonadherence and sexual dysfunction among young adult males: the cross-sectional YAMAN study. World J Urol. (2024) 42:295. doi: 10.1007/s00345-024-05003-3 38709300

[59] Wang Q Xu Z Chen X Liu L Liu X. Effect of antidepressants on ejaculation dysfunction in patients with depression and anxiety: A systematic review and network meta-analysis. Andrology. (2024). doi: 10.1111/andr.13770 39344496

[60] Dolcetti E Annovazzi P Clerico M Cocco E Conte A Marfia GA. Disease-modifying symptomatic treatment (DMST): the potential role of vortioxetine in the treatment of depression in patients with multiple sclerosis. Curr Neuropharmacol. (2024) 23. doi: 10.2174/011570159X326862240909105845 PMC12163488 39313879

[61] Di Rezze S Frasca V Inghilleri M Durastanti V Cortese A Giacomelli E. Duloxetine for the treatment of overactive bladder syndrome in multiple sclerosis. Clin Neuropharmacol. (2012) 35:231–4. doi: 10.1097/WNF.0b013e3182613dce 22751087

[62] Biyikoglu M Kettas E Sesli M Senel S Cayan S Akbay E. The effect of duloxetine on female sexual functions in the treatment of stress incontinence. Arch Gynecol Obstet. (2023) 308:1037–42. doi: 10.1007/s00404-023-07123-4 37386151

[63] Mostert JP Admiraal-Behloul F Hoogduin JM Luyendijk J Heersema DJ van Buchem MA. Effects of fluoxetine on disease activity in relapsing multiple sclerosis: a double-blind, placebo-controlled, exploratory study. J Neurol Neurosurg Psychiatry. (2008) 79:1027–31. doi: 10.1136/jnnp.2007.139345 18450787

[64] Grech LB Butler E Stuckey S Hester R. Neuroprotective benefits of antidepressants in multiple sclerosis: are we missing the mark? J Neuropsychiatry Clin Neurosci. (2019) 31:289–97. doi: 10.1176/appi.neuropsych.18070164 30945589

[65] Foley P Lawler A Chandran S Mead G. Potential disease-modifying effects of selective serotonin reuptake inhibitors in multiple sclerosis: systematic review and meta-analysis. J Neurol Neurosurg Psychiatry. (2014) 85:709–10. doi: 10.1136/jnnp-2013-306829 24403283

[66] Nathoo N Mackie A. Treating depression in multiple sclerosis with antidepressants: A brief review of clinical trials and exploration of clinical symptoms to guide treatment decisions. Mult Scler Relat Disord. (2017) 18:177–80. doi: 10.1016/j.msard.2017.10.004 29141805

[67] di Giannantonio M Montemitro C Sepede G Brunetti M Baroni G Corbo M. Agomelatine effectiveness, tolerability, and impact on anhedonia in major depression. J Clin Psychopharmacol. (2019) 39:288–90. doi: 10.1097/JCP.0000000000001038 30932949

[68] Ballard ED Wills K Lally N Richards EM Luckenbaugh DA Walls T. Anhedonia as a clinical correlate of suicidal thoughts in clinical ketamine trials. J Affect Disord. (2017) 218:195–200. doi: 10.1016/j.jad.2017.04.057 28477497 PMC5515296

[69] McIntyre RS Loft H Christensen MC. Efficacy of vortioxetine on anhedonia: results from a pooled analysis of short-term studies in patients with major depressive disorder. Neuropsychiatr Dis Treat. (2021) 17:575–85. doi: 10.2147/NDT.S296451 PMC7910099 33654400

[70] Cao B Park C Subramaniapillai M Lee Y Iacobucci M Mansur RB. The efficacy of vortioxetine on anhedonia in patients with major depressive disorder. Front Psychiatry. (2019) 10. doi: 10.3389/fpsyt.2019.00017 PMC6365446 30766492

[71] Siniscalchi A Gallelli L Tolotta GA Loiacono D De Sarro G. Open, uncontrolled, nonrandomized, 9-month, off-label use of bupropion to treat fatigue in a single patient with multiple sclerosis. Clin Ther. (2010) 32:2030–4. doi: 10.1016/j.clinthera.2010.10.012 21118738

[72] Cooper JA Tucker VL Papakostas GI. Resolution of sleepiness and fatigue: A comparison of bupropion and selective serotonin reuptake inhibitors in subjects with major depressive disorder achieving remission at doses approved in the European Union. J Psychopharmacol. (2014) 28:118–24. doi: 10.1177/0269881113514878 24352716

[73] Heitmann H Biberacher V Tiemann L Buck D Loleit V Selter RC. Prevalence of neuropathic pain in early multiple sclerosis. Multiple Sclerosis J. (2016) 22:1224–30. doi: 10.1177/1352458515613643 26480924

[74] Vollmer TL Robinson MJ Risser RC Malcolm SK. A randomized, double-blind, placebo-controlled trial of duloxetine for the treatment of pain in patients with multiple sclerosis. Pain Practice. (2014) 14:732–44. doi: 10.1111/papr.2014.14.issue-8 24152240

[75] Gallagher HC Gallagher RM Butler M Buggy DJ Henman MC. Venlafaxine for neuropathic pain in adults. Cochrane Database Systematic Rev. (2015) 2017. doi: 10.1002/14651858.CD011091.pub2 PMC6481532 26298465

[76] Adamo D Calabria E Coppola N Pecoraro G Mignogna MD. Vortioxetine as a new frontier in the treatment of chronic neuropathic pain: a review and update. Ther Adv Psychopharmacol. (2021) 11. doi: 10.1177/20451253211034320 PMC8419528 34497709

[77] Ponzio M Tacchino A Vaccaro C Brichetto G Battaglia MA Messmer Uccelli M. Disparity between perceived needs and service provision: a cross-sectional study of Italians with multiple sclerosis. Neurological Sci. (2019) 40:1137–44. doi: 10.1007/s10072-019-03780-z 30810827

[78] Palm U Ayache SS Padberg F Lefaucheur JP. Non-invasive Brain Stimulation Therapy in Multiple Sclerosis: A Review of tDCS, rTMS and ECT Results. Brain Stimul. (2014) 7:849–54. doi: 10.1016/j.brs.2014.09.014 25359259

[79] Stampanoni Bassi M Mori F Buttari F Marfia GA Sancesario A Centonze D. Neurophysiology of synaptic functioning in multiple sclerosis. Clin Neurophysiol. (2017) 128:1148–57. doi: 10.1016/j.clinph.2017.04.006 28511127

[80] McClintock SM Reti IM Carpenter LL McDonald WM Dubin M Taylor SF. Consensus recommendations for the clinical application of repetitive transcranial magnetic stimulation (rTMS) in the treatment of depression. J Clin Psychiatry. (2018) 79:35–48. doi: 10.4088/JCP.16cs10905 PMC5846193 28541649

[81] Lefaucheur JP Antal A Ayache SS Benninger DH Brunelin J Cogiamanian F. Evidence-based guidelines on the therapeutic use of transcranial direct current stimulation (tDCS). Clin Neurophysiol. (2017) 128:56–92. doi: 10.1016/j.clinph.2016.10.087 27866120

[82] Abdelhalim DS Ahmed MM Hussein HA Khalaf OO Sarhan MD. Burden of care, depression, and anxiety among family caregivers of people with dementia. J Prim Care Community Health. (2024) 15. doi: 10.1177/21501319241288029 PMC11450568 39344982

[83] McGuigan K Laurente G Christie A Carswell C Moran C Yaqoob MM. Effectiveness of interventions for informal caregivers of people with end-stage chronic illness: a systematic review. Syst Rev. (2024) 13:245. doi: 10.1186/s13643-024-02641-x 39342397 PMC11438131

